# Combining fecal microbiome and metabolomics reveals diagnostic biomarkers for esophageal squamous cell carcinoma

**DOI:** 10.1128/spectrum.04012-23

**Published:** 2024-03-18

**Authors:** Mingjun Gao, Jun Wu, Siding Zhou, Yong Chen, Mengmeng Wang, Wenbo He, Lei Jiang, Yusheng Shu, Xiaolin Wang

**Affiliations:** 1 Dalian Medical University, Dalian, China; 2 Clinical Medical College of Yangzhou University, Yangzhou, China; 3 Department of Thoracic Surgery, Northern Jiangsu People’s Hospital, Yangzhou, China; Agroscope, Nyon, Switzerland

**Keywords:** esophageal squamous cell carcinoma, gut microbiome, metabolomics, 16S rRNA amplicon sequencing, biomarkers

## Abstract

Esophageal squamous cell carcinoma (ESCC) is one of the most predominant subtypes of esophageal cancer. The characteristics of the gut microbiome and its metabolites from patients with ESCC have not been adequately studied and discussed. In this study, 40 fecal samples (20 from ESCC patients and 20 from healthy controls) were analyzed by 16S rRNA gene sequencing and untargeted metabolomics. The data sets were analyzed individually and synthesized using various bioinformatics methods. Alpha and beta diversity indicated significant differences in microbial diversity and abundance between ESCC and healthy control feces. At the genus level, the abundance of *Phascolarctobacterium*, *Sutterella*, and *Streptococcus* was significantly increased in ESCC. At the genus level, linear discriminant analysis effect size identified two biomarkers: *Bacteroides_stercoris* and *Prevotella_copri*. Untargeted metabolomics analysis revealed 307 differential metabolites between ESCC and healthy control feces, with indoles and derivatives, tropane alkaloids, lipids, and lipid-like molecules in higher relative abundance in ESCC feces than in healthy control feces. Kyoto Encyclopedia of Genes and Genomes enrichment analysis revealed that unsaturated fatty acids (FAs), ascorbate and aldarate metabolism, and hypoxia-inducible factor 1 signaling pathway were significantly associated with differential metabolite. Phenylethanolamine and despropionyl p-fluoro fentanyl could be used as reliable biomarkers to differentiate ESCC from healthy control. The correlation analysis showed that *Prevotella* may be involved in the synthesis of fatty acyl, carboxylic acids and derivatives, benzenes and substituted derivatives, organic oxygenates, and indoles and derivatives as metabolites. *Fusicatenibacter* and *Lachnospira* may be involved in the degradation of indoles and derivatives. *Alistipes*, *Agathobacter*, and *Parabacteroides* may be involved in the synthesis of indoles and derivatives with strong contributions. There is an intricate relationship between the gut microbiome and the levels of several metabolites (e.g., fatty acyls, carboxylic acids and derivatives, indoles, and derivatives). Microbial-associated metabolites can be used as diagnostic biomarkers in therapeutic exploration. Further analysis revealed that *Prevotella*, *Alistipes*, *Agathobacter*, and *Parabacteroides* might promote ESCC by regulating the synthesis of indoles and their derivatives. The results of this study provide favorable evidence for the early diagnosis of ESCC and subsequent individualized treatment and targeted interventions.

**IMPORTANCE:**

We describe for the first time the differences in fecal microbiome composition and metabolites between patients with esophageal squamous cell carcinoma (ESCC) and healthy controls by 16S rRNA gene sequencing and untargeted metabolomics. The results of this study provide a favorable basis for the early diagnosis of ESCC and subsequent targeted interventional therapy.

## INTRODUCTION

Esophageal cancer (EC) is one of the deadliest malignancies in the world, causing more than 250,000 deaths in China each year (1). Approximately 90% of esophageal cancer cases are squamous esophageal carcinoma. The early symptoms of esophageal cancer are not obvious, and most patients are advanced at the time of initial diagnosis. Although surgical resection remains the first choice of local cancer treatment, the prognosis of esophageal cancer remains poor, with a 5-year survival rate of only 20% (1). Currently, the diagnosis of esophageal squamous carcinoma mainly relies on gastrointestinal endoscopy and barium swallow, which are less sensitive and efficient (2). Therefore, for the early diagnosis and screening of esophageal cancer, there is an urgent need to develop noninvasive, highly sensitive, and specific biological markers.

It has been demonstrated that the gut microbiota play a crucial role in maintaining human health and also in some diseases, such as obesity-related diseases, liver disease, inflammatory bowel disease, and some cancers (3). Regarding the microbial characteristics of esophagus-related diseases, Deng et al. (4) performed 16S rRNA gene sequencing of feces from patients with esophageal cancer and a healthy control (HC) population and identified *Lachnospira*, *Bacteroides*, *Streptococcus*, and *Bifidobacterium* as microbiota biomarkers of esophageal cancer. In another study of esophageal squamous cell carcinoma (ESCC) patients (5) (*n* = 18), a reduction in the number of *Bacteroidetes, Fusobacteria*, and *Spirochetes* was observed. Cheung et al. (6) found enrichment of *Butyricimonas*, *Veillonella*, and *Streptococcus* in the intestinal microbiota of ESCC patients and depletion of *Butyrospermopsis*, *Lachnospiraceae* NK4A136 group, and *Eubacterium eligens*. Meng et al. found that *Porphyromonas gingivalis* in the saliva of ESCC patients induces ESCC tumorigenesis through the NF-κB signaling pathway (7). Different intestinal flora of ESCC patients were found in these studies. Therefore, there is a need to further investigate the relationship between ESCC and intestinal flora.

Recent studies have shown that metabolomics plays a significant role in the pathogenesis and biomarker prediction of ESCC. Using metabolomics, Zhu and colleagues analyzed plasma metabolites in 140 ESCC patients and 170 healthy controls, found significant differences between the two populations, and constructed a validated ESCC diagnostic model based on eight metabolites (8). In addition, Zhu et al. screened a set of markers consisting of 3′-UMP, palmitaldehyde, palmitoleic acid, and isobutyl decanoate for predicting ESCC recurrence with an area under the curve (AUC) of 0.98 by analyzing plasma exosome-targeted metabolomics in ESCC patients and comparing patients with recurrence to those without recurrence (9). In another study, 11 metabolites were identified as potential therapeutic biomarkers by comparing patients with ESCC before and after treatment, and the levels of three of them [octanoyl carnitine, decanoylcarnitine, and lysoPC (16:1)] were strongly correlated with treatment outcome (10).

Gut metabolites, as messengers between the gut microbiota and host cells, are key mediators in the inter-regulation of the human body and microbiota, influencing host health and disease development (11). However, the relationship between gut microbes and their metabolites and ESCC has not been adequately studied. Therefore, we analyzed the gut flora structure using 16S rRNA gene sequencing in preoperative patients with esophageal squamous carcinoma and sex- and age-matched healthy controls, and we also performed an untargeted metabolomics study based on Liquid Chromatograph Mass Spectrometer (LC-MS) technology to analyze the gut microbiota from primary metabolites in stool samples from clinical patients with ESCC and healthy controls. In this way, we investigated the mechanism of the gut microbiome and its metabolites on ESCC, screened potential biological markers with characteristics, and provided effective evidence for early prevention of EC.

## RESULTS

### Gut microbial profiles

16S rRNA gene sequencing obtained a median of 99,930 clean reads for the stools of 20 ESCC patients and 20 matched healthy controls (Table S2). After categorical assignment, a total of 1,597 operational taxonomic units (OTUs) were obtained for the ESCC group and HC, with a total of 912 OTUs for the ESCC group and 1,470 OTUs for the HC group (Fig. S1A). The species accumulation boxplot (Fig. S1B) and (rarefaction curve; Fig. S1C) of all samples indicated the reliability of our study.

**Fig 1:**
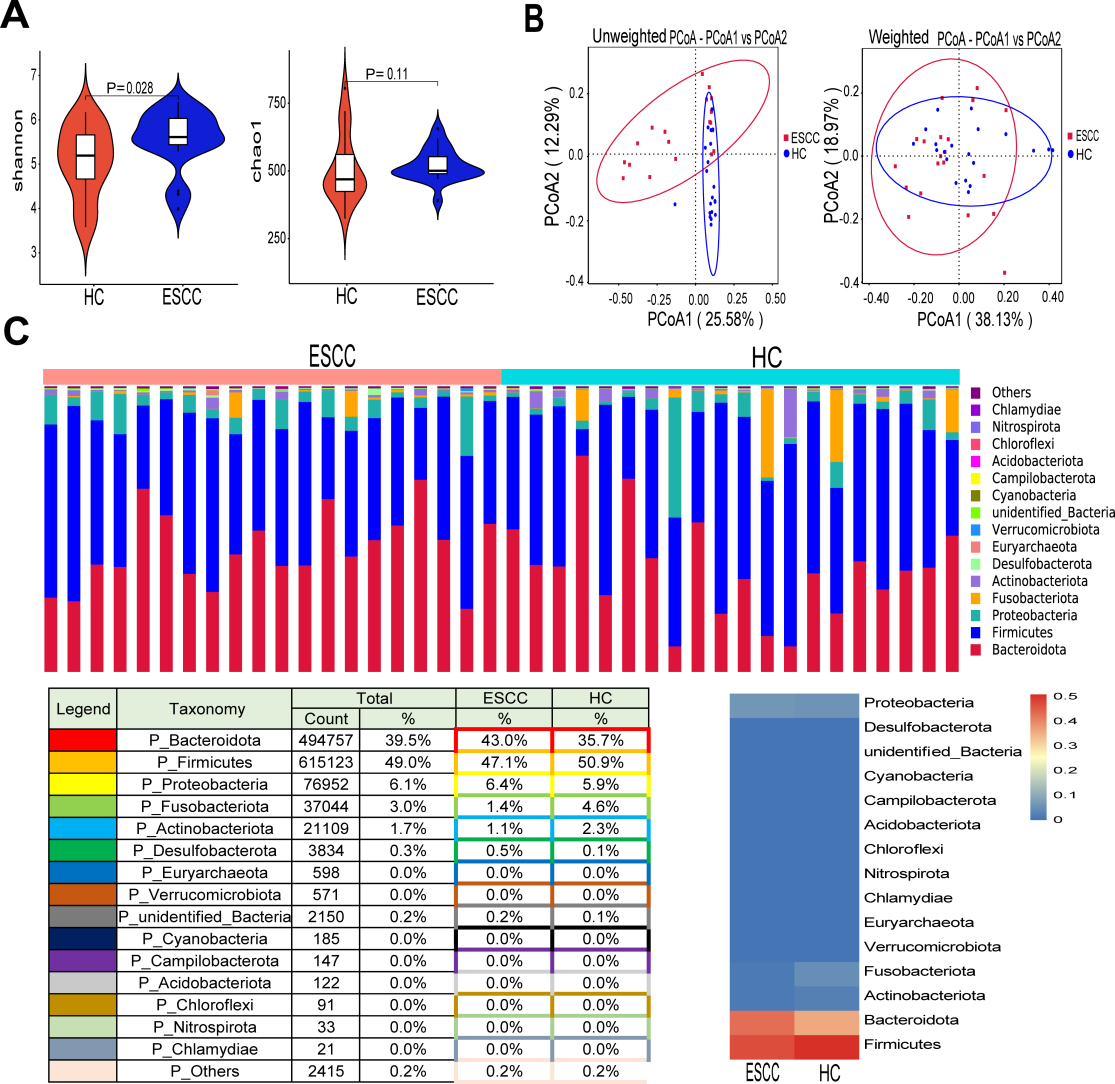
Diversity and structural analysis of the gut microbiome in esophageal squamous carcinoma. (**A**) Differences in alpha diversity between ESCC and HC for Shannon, chao1 index. (**B**) Beta diversity differences between ESCC and HC were estimated by PCoA. Left, unweighted PCoA plots; Right, weighted PCoA plots. ESCC group (red dots); HC group (blue dots); each dot represents a single sample. (**C**) Proportions of bacterial phylum levels in ESCC and HC groups. ESCC group: *n* = 20 and HC group: *n* = 20. Bottom left: relative proportions of dominant taxa at the phylum level assessed by assignment of microbial taxa. Bottom right: heat map showing the relative abundance of bacterial phylum in the two groups. The rows indicate each bacterial phylum, and the relative abundance is indicated by the color gradient.

Alpha diversity was analyzed to examine differences in microbial diversity between groups. Shannon’s diversity index was plotted against each other, reflecting the differences in species homogeneity and diversity between the two subgroups (Fig. 1A). The Shannon’s index was higher in ESCC than in HC (*P* = 0.0283). While chao1 (*P* = 0.114) was not significantly different between the ESCC and HC groups (Fig. 1A). Furthermore, principal coordinate analysis (PCoA) of Beta diversity was performed, and both unweighted and weighted PCoA plots showed the presence of its unique microecological structure in the ESCC group (Fig. 1B).

### Alterations in the composition of gut microflora associated with esophageal squamous cell carcinoma

At the phylum level of microbial composition, we found that the top six relative abundances in the ESCC group were in phyla *Firmicutes* (47.1%), *Bacteroidota* (43.0%), *Proteobacteria* (6.4%), *Fusobacteriota* (1.4%), *Actinobacteriota* (1.1%), and *Desulfobacterota* (0.5%; Fig. 1C). We further analyzed the distribution of intestinal flora at the genus level in both groups of samples. The top five relative abundances in the cancer group were *Bacteroides* (20.69%), *Prevotella* (15.96%), *Faecalibacterium*(8.30%), *Blautia* (3.74%), and *Parabacteroides* (2.93%), respectively (Fig. S2A and B).

To compare the differences in fecal flora between the two groups, Welch’s *t* test was performed for different taxonomic levels. At the phylum level, the abundance of *Desulfobacterota* (*P* = 0.0061) was significantly higher in the ESCC group than in the HC group. At the genus level, a total of 48 genera were significantly different between the two groups (Table S3). It was found by the *t*-test that the cancer group had a higher proportion of *Phascolarctobacterium*, *Sutterella*, and *Streptococcus* compared to the healthy group, while the proportions of *Lachnospira* and *Parasutterella* were significantly lower, with statistical differences (*P* < 0.05; Fig. S1E).

**Fig 2:**
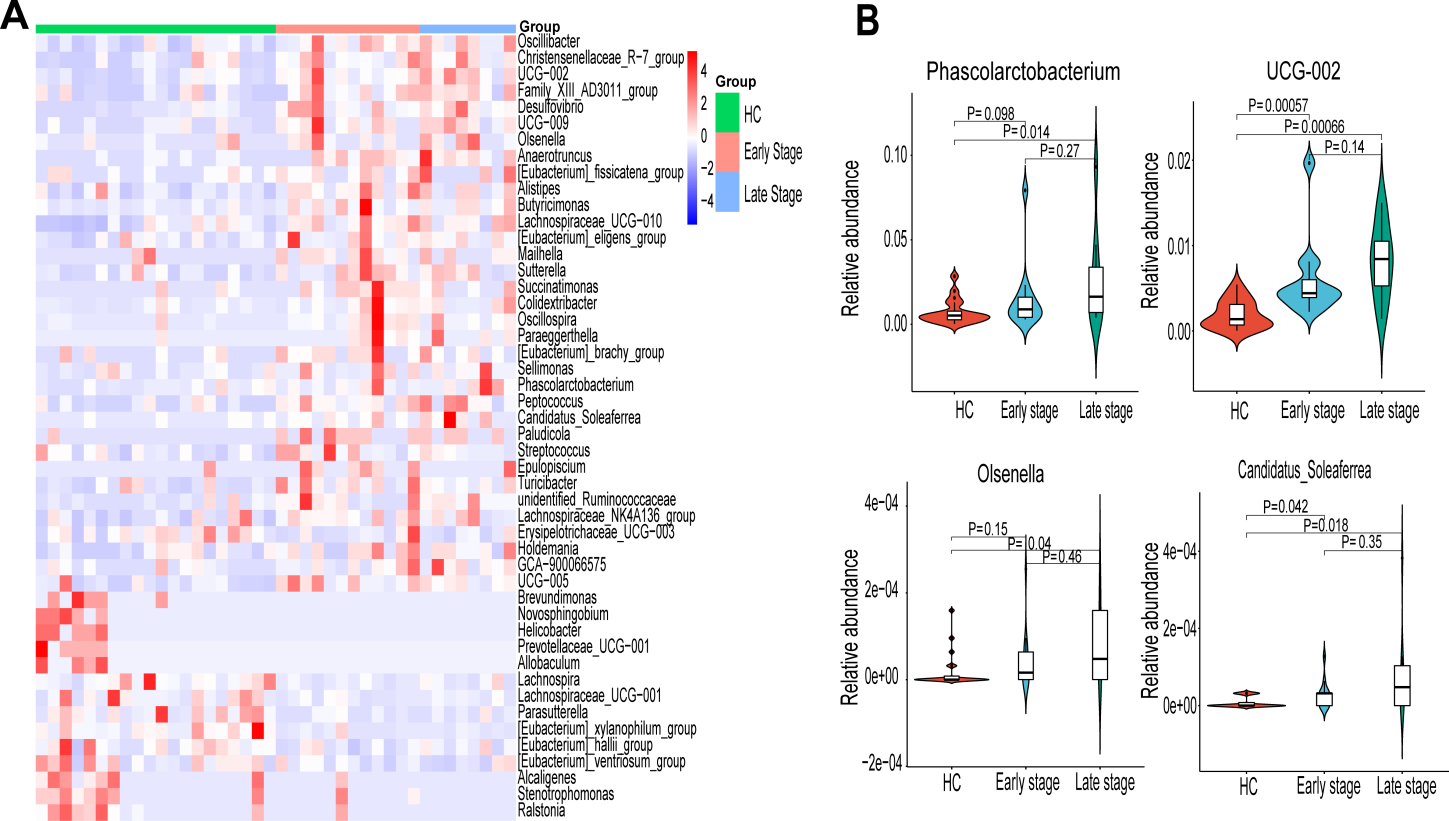
The heat map shows the association between the 48 significantly different genera and tumor stages. (**A**) Showing differences in flora between HC (*n* = 20), early (stages I–II, *n* = 12), and late ESCC (stages III–IV, *n* = 8). The heat map shows the relative abundance of the 48 differentially significant genera. (**B**) The relative abundance of *Phascolarctobacterium*, UCG-002, *Olsenella*, and *Candidatus_Soleaferrea* varied between HC, early, and late ESCC.

Twelve patients with early (stages I–II) and eight patients with advanced (stages III–IV) ESCC were included in this study (Table S1). The relative abundance heat map of the 48 genera is shown in Fig. 2A. Overall, there were 34 OTUs in the ESCC group and 14 OTUs in the HC group. (Table S3). A gradual increase in the abundance of *Phascolarctobacterium*, *UCG-002*, *Olsenella*, and *Candidatus_Soleaferrea* was observed from the healthy population to patients with early and advanced ESCC (Fig. 2B). However, the differences were not significant (*P* > 0.05).

### Analysis of the differential taxa between ESCC and HC

**Fig 3:**
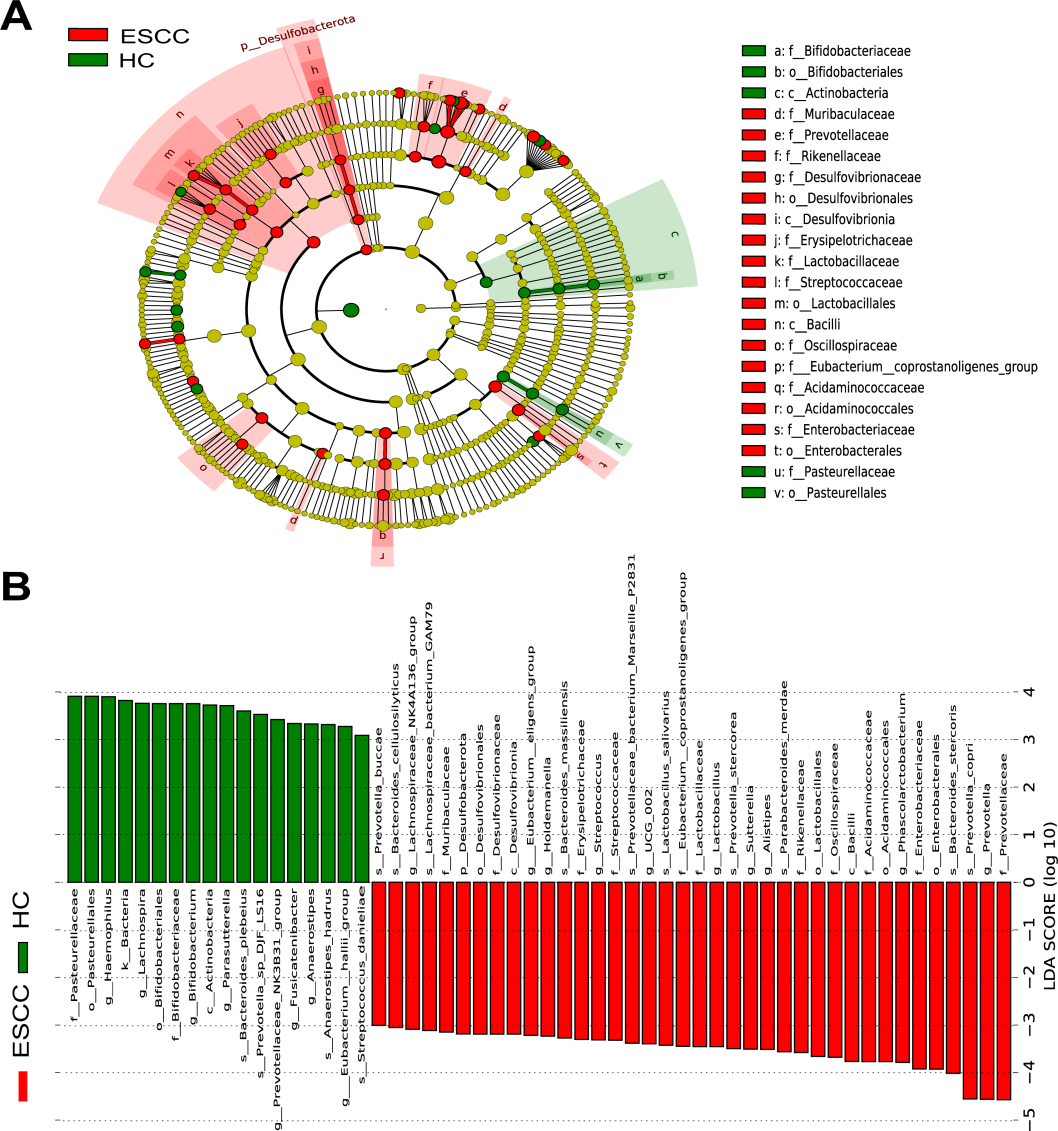
Linear discriminant analysis (LDA) was combined with effect size (LEfSe). (**A**) Taxa of microbiota associated with ESCC and HC groups. (**B**) Histogram of LDA scores for ESCC and HC groups, with each microbiota LDA score indicating the effective size of its differential abundance taxonomic unit (LDA > 3).

Using the linear discriminant analysis (LDA) effect size (LEfSe) method to analyze the composition of the intestinal flora of ESCC and HC, we found that 56 OTUs (LDA > 3) were significantly different (Fig. 3), with *s_Bacteroides_stercoris*, *s_Prevotella_copri*, *f_Prevotellaceae*, and *g_Prevotella* (all LDA scores (log10) >4) were more abundant in the ESCC group than in the HC group.

Contrarily, *Pasteurellaceae*, *Haemophilus*, *Lachnospira*, *Bacteroides_plebeius*, *Bifidobacterium*, *Parasutterella*, and *Prevotellaceae_NK3B21_group* were significantly more abundant in the HC group [all LDA scores (log10) >3.5] than ESCC group (Fig. 3).

To characterize the functional alterations in the gut microbiota of ESCC patients, we predicted the functional composition profile using 16S rRNA sequencing data from Tax4Fun analysis (Fig. S3).

### Differences in fecal metabolomic profiles between ESCC and healthy controls

**Fig 4:**
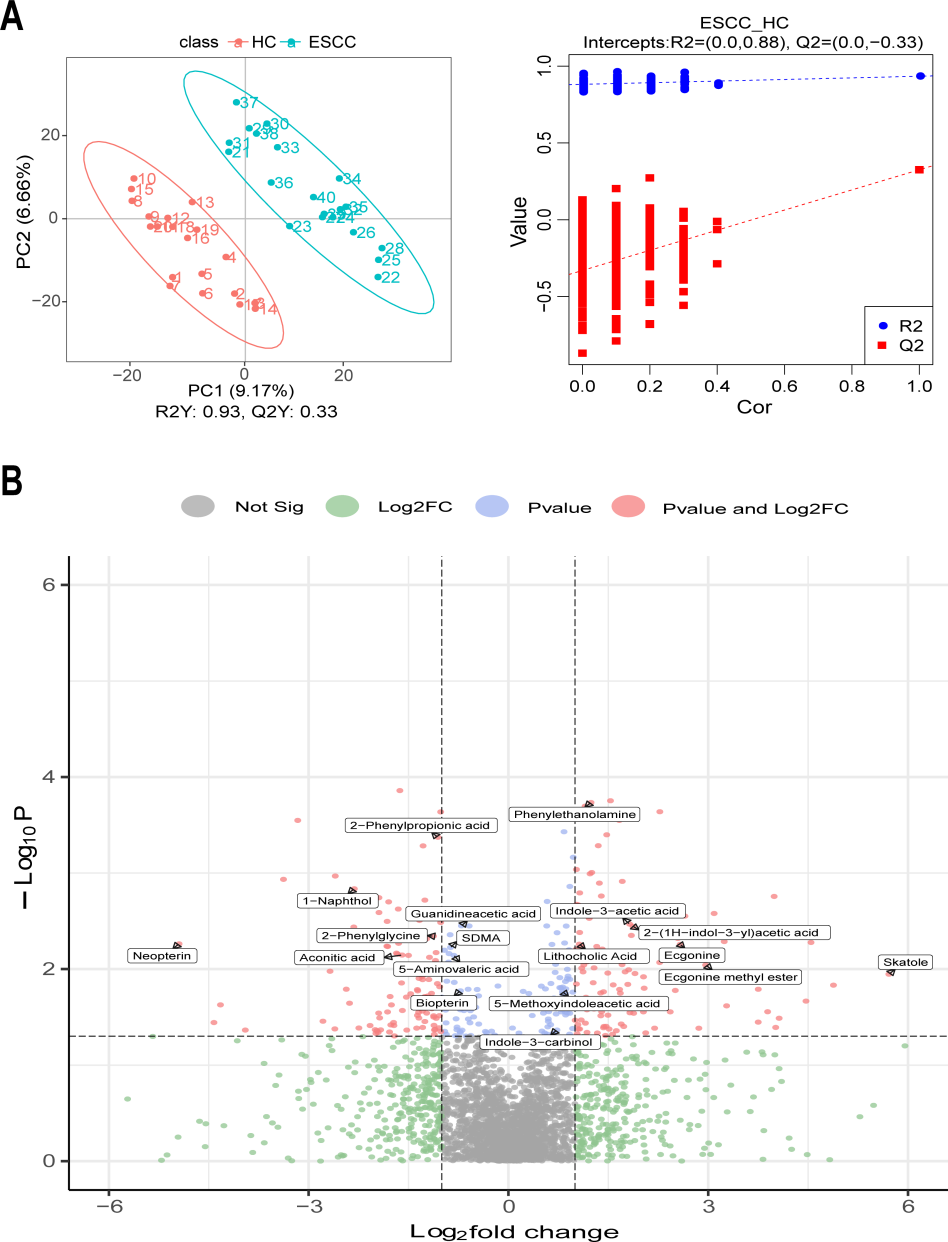
Differential metabolites in ESCC and HC. (**A**) The PLS-DA results showed that ESCC and HC were divided into two different clusters, and the test of the PLS-DA model showed that the present study was valid. (**B**) Volcano plot of differential metabolites between the ESCC and HC groups (VIP > 1 and *P*-value < 0.05).

The relative quantitative values of metabolites were used to calculate the Pearson correlation coefficient between quality control samples, and the closer the R^2^ to 1, the higher the correlation, the more stable the whole assay process, and the more accurate and reliable the data results (Fig. S4A). Principal component analysis reflected the overall metabolic differences in the samples and the magnitude of variability between the groups (Fig. S4B). The partial least squares discriminant analysis (PLS-DA) score plot showed that ESCC and HC were divided into two different clusters (R2Y = 0.93 and Q2Y = 0.33). Tests of the PLS-DA model showed that R2 values were greater than Q2 values, and the Q2 regression line had a negative intercept [R2 = (0.0, 0.88) and Q2 = (0.0, –0.33)], indicating that the PLS-DA model of this study was valid (Fig. 4A). Volcano plots were used to depict 307 metabolites with significant differences in relative abundance between ESCC and HC [variable importance in projection (VIP) >1 and *P*-value < 0.05; Fig. 4B], 145 differential metabolites were down-regulated, and 162 were up-regulated in ESCC compared to HC (Table S4). In the ESCC group, the abundant metabolites were indoles and derivatives [indole-3-acetic acid, 2-(1H-indol-3-yl)acetic acid, skatole, 5-methoxyindoleacetic acid, and indole-3 carbinol]; tropane alkaloids (ecgonine and ecgonine methyl ester); lipids and lipid-like molecules (lithocholic acid); compared to the HC group which showed higher levels of carboxylic acids and derivatives (guanidineacetic acid, 2-Phenylglycine, Symmetric dimethylarginine (SDMA), aconitic acid, and 5-Aminovaleric acid); naphthalenes (1-Naphthol); phenylpropanoic acids (2-Phenylpropionic acid); pteridines and derivatives (neopterin and biopterin).

### Multiple analytical methods reveal discriminatory metabolites between the ESCC and HC groups

**Fig 5:**
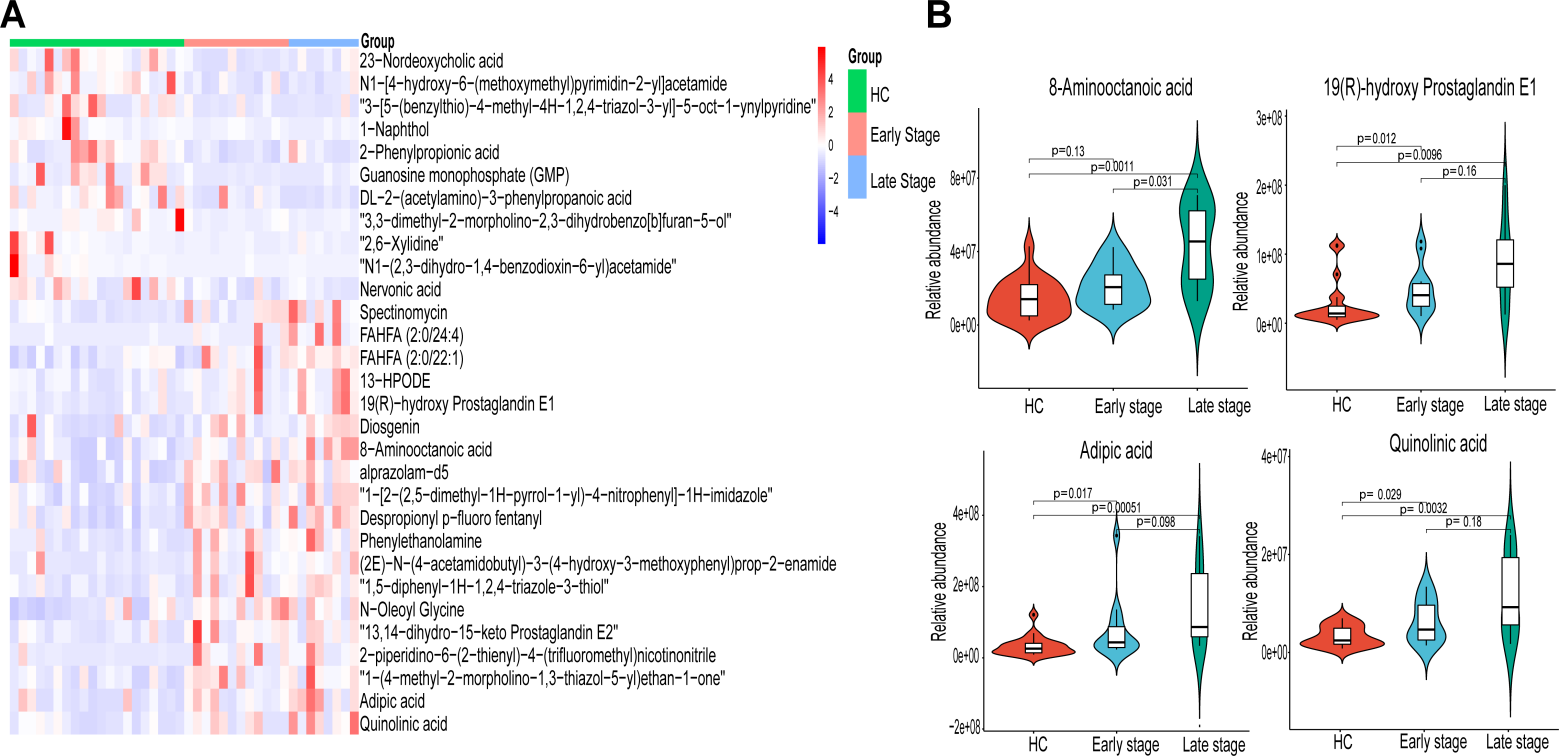
The heat map shows the association between the 30 significantly different metabolites and tumor stage. (**A**) Showing differences in flora between HC (*n* = 20), early (stages I–II, *n* = 12), and late ESCC (stages III–IV, *n* = 8). The heat map shows the relative abundance of the 30 significantly different metabolites. (**B**)The relative abundance of 8-Aminooctanoic acid, 19(R)-hydroxy Prostaglandin E1, Adipic acid, and Quinolinic acid varied between HC, early, and late ESCC.

Clustering, correlation, and multivariate analysis revealed differential metabolites between ESCC and HC groups, with hierarchical clustering of the top 30 significantly differential metabolites (Fig. 5A). A gradual increase in the abundance of 8-Aminooctanoic acid, 19(R)-hydroxy Prostaglandin E1, adipic acid, and quinolinic acid was observed from the healthy population to patients with early and advanced ESCC (Fig. 5B). Among them, 8-Aminooctanoic acid was significantly higher in patients with advanced ESCC (*P* < 0.05).

**Fig 6:**
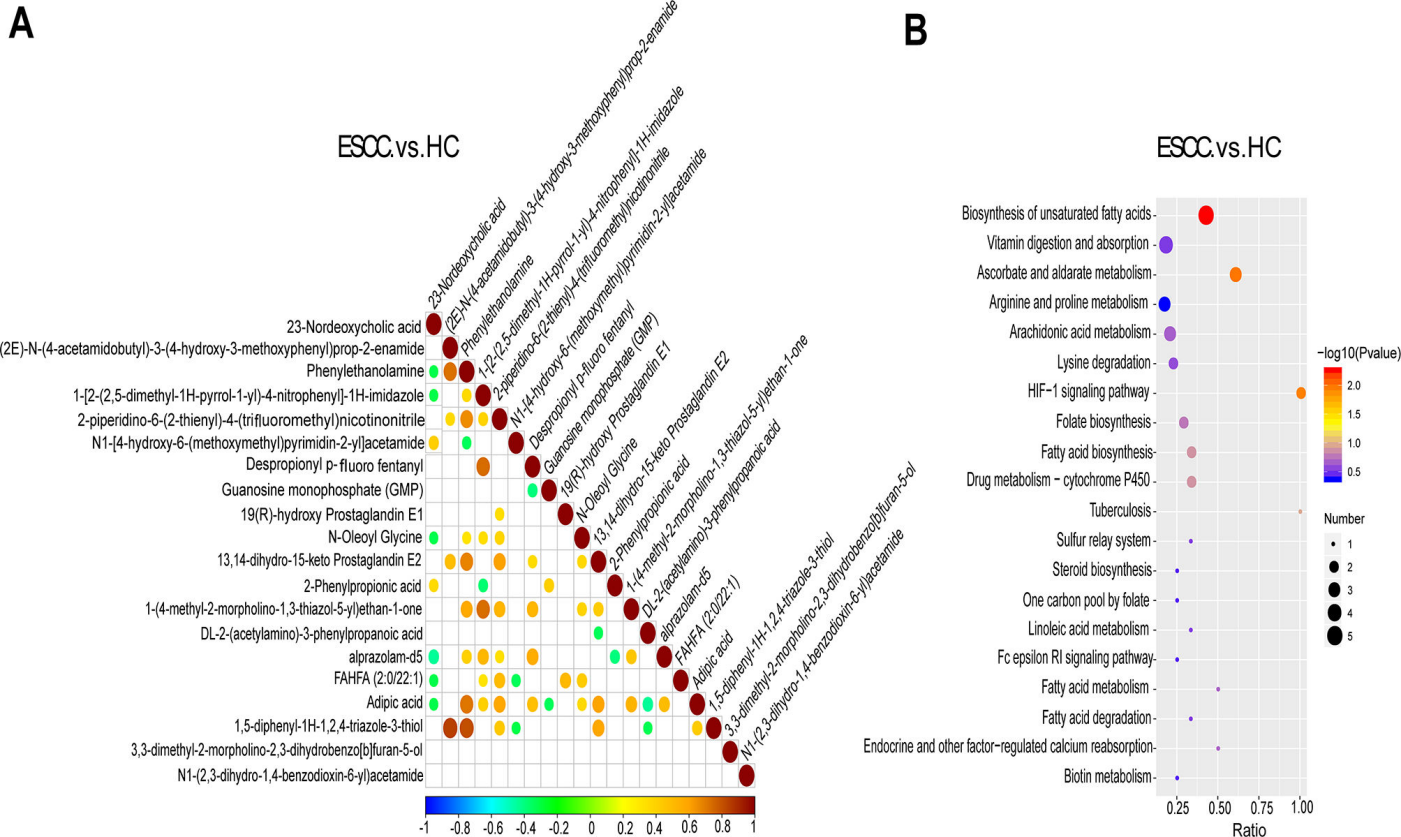
Differential metabolite correlation analysis and KEGG analysis. (**A**) The Pearson correlation coefficients were calculated between all metabolites. Complete positive correlations are indicated in red in the figure, and complete negative correlations are indicated in blue in the figure. The figure shows the correlations of the top 20 differential metabolites ordered by *P*-value from smallest to largest. (**B**) KEGG enrichment scatter plot showing biological processes and functions of fecal differential metabolites between ESCC and HC groups.

The expression and relationship of different metabolites between different samples were explored (Table S5), showing the correlation of the top 20 differential metabolites (Fig. 6A). Kyoto Encyclopedia of Genes and Genomes (KEGG) enrichment analysis was performed to identify the major metabolic pathways and signaling pathways associated with differential metabolites in healthy and tumor populations (Table S6). KEGG enrichment bubble map results showed that the biosynthesis of unsaturated fatty acids (five differential metabolites), ascorbate and aldarate metabolism (three differential metabolites), and hypoxia-inducible factor 1 (HIF-1) signaling pathway (two differential metabolites) showed a high correlation with differential metabolites, *P* < 0.05 (Fig. 6B).

### Identification of metabolite biomarkers to distinguish ESCC and HC

**Fig 7:**
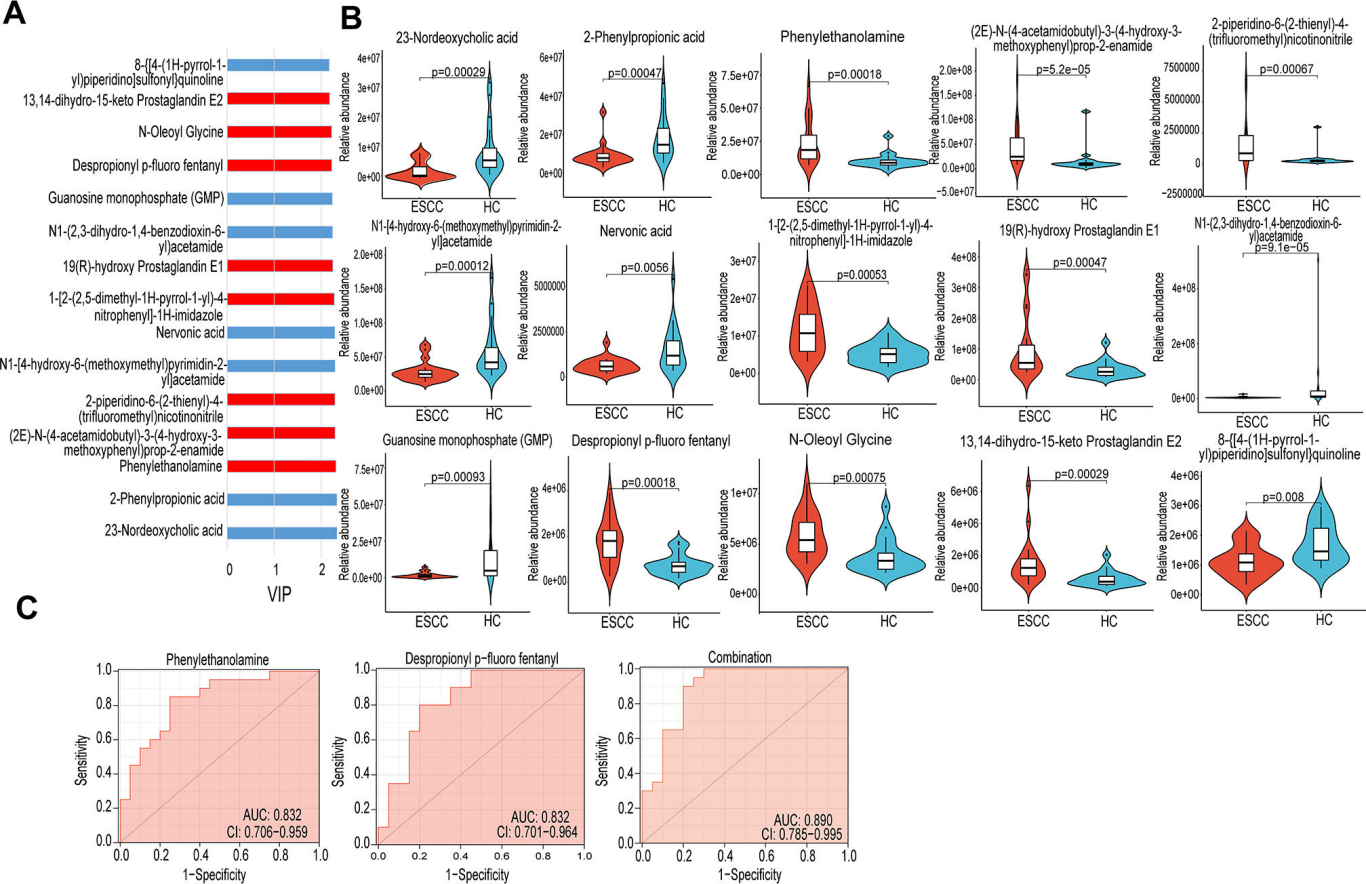
ESCC and HC differential metabolites as biomarkers. (**A**) Shows the top 15 metabolites ranked according to VIP value. (**B**) Of the 15 metabolites, eight metabolites have high relative abundance in ESCC. (**C**) ROC analysis of Phenylethanolamine and Despropionyl p-fluoro fentanyl and combinations of the two metabolites.

To identify metabolite biomarkers used to differentiate tumor and non-tumor tissues, we selected the top 15 metabolites based on their VIP values (Fig. 7A). Among the 15 metabolites, the relative abundance of eight metabolites was higher in patients with esophageal squamous carcinoma than in healthy controls (Fig. 7B). Next, we performed receiver operating characteristic curve (ROC) analysis and obtained two candidate biomarkers, phenylethanolamine and despropionyl p-fluoro fentanyl. They corresponded to an AUC of 0.832 (95% CI: 0.706–0.959) and 0.832 (95% CI: 0.701–0.964; Fig. 7C). The AUC for the combination of the two metabolites was 0.890 (95% CI: 0.785–0.995; Fig. 7C). These results suggest that the combination of phenylethanolamine and despropionyl p-fluoro fentanyl may serve as a potential biomarker to differentiate ESCC from the healthy population.

### The relationship between discriminative genera and metabolites in different classes

**Fig 8:**
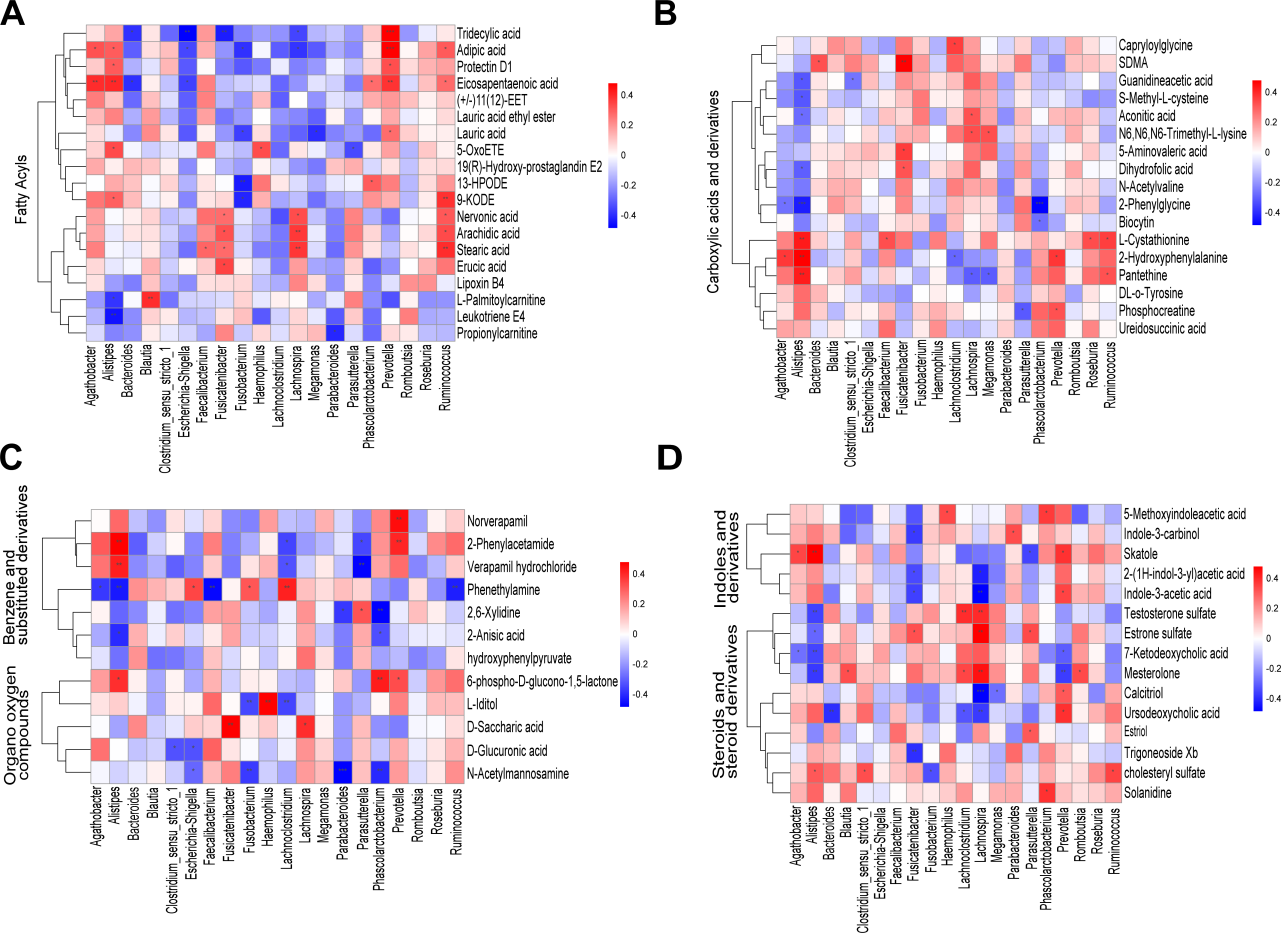
Correlation analysis of different classes of differential genera and metabolites (**A–D**) The analysis of the correlation between 20 differential genera and discriminative metabolites in the classes of (**A**) fatty acyls (*n* = 19), (**B**) carboxylic acids and derivatives (*n* = 17), (**C**) benzene and substituted derivatives (*n* = 7) and organooxygen compounds (*n* = 5), and (**D**) indoles and derivatives (*n* = 5), steroids and steroid derivatives (*n* = 10). Taxonomic annotation of metabolites based on Human Metabolome Database. Red, positive correlation; blue, negative correlation. *P value < 0.05; **P value < 0.01.

The analysis of the association between differential genera and metabolites in different classes was performed. As shown in Fig. 8, *Prevotella* was positively correlated with the majority of differential metabolites in the classes of fatty acyls, carboxylic acids and derivatives, benzene and substituted derivatives, organooxygen compounds, and indoles and derivatives. These results indicated that *Prevotella* might contribute to the synthesis of metabolites in these classes. *Alistipes* exhibited a significant association with seven fatty acyls and eight carboxylic acids and derivatives, while *Faecalibacterium* showed a significant correlation with six fatty acyls and two carboxylic acids and derivatives (Fig. 8A and B). These results suggested that *Alistipes* and *Faecalibacterium* might play an important role in the synthesis or degradation of fatty acyls and carboxylic acids and derivatives. *Fusicatenibacter* and *Lachnospira* showed a significantly negative association with indoles and derivatives, indicating a robust contribution of *Fusicatenibacter* to the degradation of indoles and derivatives (Fig. 8D). While *Alistipes*, *Agathobacter*, and *Parabacteroides* showed a significantly positive association with indoles and derivatives, indicating a robust contribution of *Fusicatenibacter* to the synthesis of indoles and derivatives. *Ruminococcus* was significantly positively associated with six fatty acyls and two carboxylic acids and derivatives, which suggested that *Ruminococcus* might participate in the synthesis of carbohydrates (Fig. 8A and B). We further mapped the association network of correlations between differential microorganisms and metabolites (Fig. S5).

## DISCUSSION

To our knowledge, this is the first study to explore the differences in the diversity of fecal microorganisms and their metabolites between ESCC patients and healthy populations. In this paper, the diversity and abundance of gut microbiota were significantly different between ESCC and HC, which is consistent with the results of previous studies (4). At the phylum level, *Bacteroidota*, *Proteobacteria*, and *Desulfobacterota* were significantly higher in the ESCC group. *Firmicutes*, *Fusobacteriota*, and *Actinobacteria* were significantly lower in the ESCC group. However, Deng et al. (4) observed that at the phylum level, the EC group showed a higher abundance of *Firmicutes* and *Actinobacteria* but lower than HC for *Actinobacteriotax*. There is no consensus about the relationship between gut microbial diversity and ESCC.

We used the LEfSe method to find *Bacteroides* and *Prevotellas* as the most significantly different flora. *Prevotella* and *Anaplasma* may serve as valid biomarkers of diet and lifestyle (12). In studies, *Prevotella* is associated with a non-polysaccharide and fiber-rich plant-based diet (13, 14), while the *Bacteroides* enterotype is usually associated with a diet rich in protein and fat (15). In one study, *Prevotella* was found to be significantly associated with a better prognosis for colorectal cancer, and the genus *Mycobacterium* was associated with a worse prognosis (16). Pei et al. found a predominance of the phylum *Synechococcus* and the genus *Prevotella* in normal esophageal tissue biopsies (17). Jiang et al. compared healthy control, esophagitis, and ESCC samples and found a significant decrease in *Mycobacterium avium* and an increased abundance of *Prevotella* in ESCC (18). However, Yang et al. (5) demonstrated significant enrichment of *Prevotella* and *Bacteroides* in ESCC. This is consistent with our findings, where we found significantly elevated fecal *Bacteroides* and *Prevotellas* in ESCC patients. Thus, *Bacteroides* and *Prevotellas* may have a bidirectional role in tumorigenesis, and their role in ESCC needs to be further investigated in the future.

Our metabolomic analysis of ESCC feces and those of healthy controls revealed 307 differential metabolites, including carboxylic acids and derivatives, benzene and substituted derivatives, fatty acyls, indoles and derivatives [indole-3-acetic acid, 2-(1 h-indol-3-yl)acetic acid, skunk, 5-methoxyindoleacetic acid, and indole-3 methanol], which were significantly elevated in the fecal metabolites of ESCC patients. Indole derivatives are processed by intestinal bacteria on dietary tryptophan and act by binding to the aryl hydrocarbon receptor (AHR) on cells (19). AHR acts as a cytosolic ligand-activated transcription factor and plays a crucial role in maintaining cellular homeostasis (20). Numerous studies have shown that the physiological effects of AHR activation play a key role in tumorigenesis and immune regulation, and Mo et al. studied the gene expression data and clinical characteristics of 33 human cancers through the cancer genome database as a way to assess the prognostic value of AHR and found a strong correlation between AHR and immune cell infiltration and immune regulation, and high expression of AHR was significantly associated with immune-related pathways (21). Scott et al. demonstrated that tryptophan metabolites derived from the gut microbiota (indole-3-ethanol, IPyA, and I3A) could modulate the barrier function of the gut via the AHR in some inflammatory stimuli, including pro-inflammatory cytokines and dextran sulfate sodium (DSS) colitis (22). Activation of the AHR is influenced by diet and gut microbial composition. For example, specific *Lactobacillus* metabolizes tryptophan from food to form indole derivatives activating AHR receptors to produce IL-22, providing colonization resistance to the fungus *Candida albicans* and mucosal protection from inflammation (23). In addition, AHR and its ligands (agonists or antagonists) are expected to become new targets for blocking cancer (24). Our data found that all five indole derivatives in differential metabolism were upregulated in the T group, demonstrating that indole derivatives may play an important role in ESCC development.

KEGG enrichment analysis showed that unsaturated fatty acid biosynthesis, ascorbate and aldehyde metabolism, and HIF-1 signaling pathway showed a high correlation with differential metabolites (*P* < 0.05). FAs are the major component of several lipids, including phospholipids, triglycerides, and sphingolipids, and further synthesize more complex lipids, including triglycerides and diglycerides, through various metabolic pathways (25). The role of FA metabolism, including uptake, ab initio synthesis, and oxidation of FAs, on cancer cells includes induction of angiogenesis, epithelial-mesenchymal transition, involvement in apoptosis and autophagy, and maintenance of proliferative signals (26). As one of the lipid metabolic pathways, fatty acid synthesis converts nutrients into metabolic intermediates for energy storage, biofilm synthesis, and signaling molecule production (27). There has been extensive research on fatty acid metabolism and cancer. Chu et al. performed the metabolomic analysis of normal and cancerous breast tissues and identified the fatty acid receptor FFAR4 expression as a prognostic biomarker in patients with hormone receptor-positive breast cancer treated with tamoxifen. FFAR4 could be a novel potential target for anti-breast cancer therapy, especially for patients with endocrine resistance (28). Yu et al. identified genetic variants in the fatty acid biosynthesis pathway that play an important role in colorectal cancer outcomes (29). Our findings revealed a significant correlation between the biosynthesis of unsaturated fatty acids and differential metabolites in differential metabolite enrichment analysis. Zhu et al. observed significant metabolic reprogramming in esophageal cancer, including glycolysis, glutamine catabolism, and fatty acid metabolism, in a metabolomic analysis of patients with early-stage esophageal cancer and a healthy group of people (30). This is consistent with the results of our study and again demonstrates that fatty acid metabolism is closely associated with ESCC. HIF-1 is a key regulator of the metabolic reprogramming that occurs in hypoxic cancer cells (31). Tang et al. found that high levels of HIF-1α expression were associated with an aggressive phenotype and poor prognosis in ESCC patients (32). It has been shown that HIF-1α is highly expressed in all ESCC tissues compared to normal esophageal tissues and maybe a poor prognostic survival factor for ESCC (33). Our study also found a significant correlation between the HIF-1 signaling pathway and ESCC.

According to Spearman’s correlation analysis, the relative abundance of differential metabolites (stearic acid, arachidic acid, eicosapentaenoic acid, erucic acid, and nervonic acid) in the biosynthetic pathway of unsaturated fatty acids was correlated with that of *Ruminococcus*. In addition, *Lachnospira* and *Fusicatenibacterc* were significantly and positively correlated with fatty acyls, demonstrating that *Ruminococcus*, *Lachnospira*, and *Fusicatenibacterc* may be jointly responsible for altering the relative abundance of metabolites in fatty acyls groups. *Faecalibacterium*, *Fusicatenibacter*, *Prevotella*, *Roseburia*, and *Ruminococcus* may be jointly responsible for the synthesis of carboxylic acids and derivatives. *Fusicatenibacter* and *Lachnospira* showed a significant negative correlation with indoles and derivatives, while *Alistipes*, *Agathobacter*, *Parabacteroides*, and *Prevotella* showed a significant positive correlation with indoles and derivatives, indicating that these microorganisms are involved in the degradation and synthesis of indoles and their derivatives. Indole acetic acid (IAA) is an intestinal bacterial-derived tryptophan metabolite. Chen et al. (34) found that IAA treatment appeared to construct a new microbial environment, leading to an increase in the proportion of *Prevotella* and *Parabacteroides* spp. These data suggest that *Prevotella* may influence the occurrence of ESCC through the regulation of indoles and derivatives.

The present study has some shortcomings. First, due to the strict entry requirements of metabolomics and the limited sample size in a single-center study, future multi-center studies are still needed to validate the results. Second, this study only used untargeted metabolomics to screen metabolic pathways and metabolic markers closely related to ESCC, further validated the results through targeted metabolomics, and cellular and animal experiments are still needed. Finally, dietary habits and medications can affect microbial composition and metabolism, but we do not have access to the dietary and medication data of patients.

In conclusion, we describe for the first time the differences in fecal microbiome composition and metabolites between patients with esophageal squamous carcinoma and healthy controls. There were significant differences in the diversity and composition of the intestinal flora between patients with esophageal squamous carcinoma and healthy controls, with significant aggregation of *Bacteroides* and *Prevotella* in ESCC. The intestinal metabolomic profile was significantly different between patients with esophageal squamous carcinoma and healthy controls, and phenylethanolamine and despropionyl p-flufentanil could be used as biomarkers to distinguish ESCC patients from normal subjects. Spearman correlation analysis revealed that *Prevotella*, *Alistipes*, *Agathobacter*, and *Parabacteroides* might be involved in indoles and derivatives, and the expression of indoles and derivatives was all increased in ESCC among the differential metabolites, demonstrating that these florae may promote ESCC by regulating the synthesis of indoles and derivatives. The results of this study contribute to the understanding of the molecular pathogenesis of ESCC through the perspective of gut microbes and metabolites and provide favorable evidence for early diagnosis of ESCC and subsequent individualized treatment and targeted interventions.

## MATERIALS AND METHODS

### Study design and stool sample collection

Twenty patients with esophageal cancer, diagnosed preoperatively by gastroscopy and pathological biopsy as esophageal squamous cell carcinoma, attended the Department of Thoracic Surgery at Northern Jiangsu People’s Hospital (Yangzhou, China) between July 2021 and December 2021 and were included in this study. Healthy controls: 20 healthy people matched with esophageal cancer patients regarding age, gender, body mass index, and dietary habits. Exclusion criteria: (i) inflammation of the gastrointestinal tract within 8 weeks; (ii) taking antibiotics, proton pump inhibitors, hormones, non-steroidal anti-inflammatory, immunosuppressive drugs, and other drugs that may affect the experimental error within the last 8 weeks; (iii) patients who had received chemotherapy, radiotherapy, immunotherapy, and targeted therapy; (iv) patients with a history of other digestive system diseases; (v) patients with metabolic diseases such as diabetes, ventilation, hyperlipidemia, and hyperthyroidism; (vi) combined with other malignant tumors; (vii) lack of basic clinical information. Clinicopathological characteristics were collected at the time of inclusion (Table S1). The study was approved by the ethics committee under the Northern Jiangsu People’s Hospital and approved by the corresponding regulatory agency. The subjects who obtained the consent signed the informed consent form with the patients and their families in person. Face-to-face interviews were conducted with the enrolled population to obtain basic information on weight, height, smoking, alcohol consumption, and diet. The stool was collected in a sterile stool sampler, and each subject was given at least three tubes of the stool of at least 1.5 g. One tube was used for 16S rRNA gene sequencing, and one tube was used for untargeted metabolomics. The collected samples were immediately snap-frozen in liquid nitrogen for 10 min to avoid degradation of the samples and then stored in a −80°C refrigerator for long-term storage.

### Fecal DNA extraction, 16S assay

The total genomic DNA of the samples was extracted by the Hexadecyl trimethyl ammonium Bromide (CTAB) (35) method. DNA concentration and purity were detected on 1% agarose gel. DNA was diluted to 1 ng/µL with sterile water according to the concentration. The primers targeting the V4 region of the 16S rRNA gene are as follows: 515F: 5′-GTGCCAGCMGCCGCGGTAA-3′ and 806R: 5′-GGACTACHVGGGTWTCTAAT-3′. All PCR reactions were carried out with 15 µL of Phusion High-Fidelity PCR Master Mix (New England Biolabs), 2 µM of forward and reverse primers, and about 10 ng template DNA. Thermal cycling consisted of initial denaturation at 98°C for 1 min, followed by 30 cycles of denaturation at 98°C for 10 s, annealing at 50°C for 30 s, and elongation at 72°C for 30 s. Finally, 72°C for 5 min. The PCR products were detected by electrophoresis. The qualified PCR products were purified by magnetic beads, quantified by enzyme labeling, mixed in equal amounts according to the concentration of the amplified products, and then recovered by gel electrophoresis after sufficient mixing. Sequencing libraries were generated usingTruSeq DNA PCR-Free Sample Preparation Kit (Illumina, USA) following the manufacturer’s recommendations, and index codes were added. The library quality was assessed on the Qubit@ 2.0 Fluorometer (Thermo Scientific) and Agilent Bioanalyzer 2100 system. Finally, the library was sequenced on the Illumina NovaSeq6000 platform to obtain 250 bp paired-end reads.

### Sequencing data analysis

The data of each sample was split from the downstream data according to the Barcode sequence and PCR amplification primer sequence, and the reads of each sample were spliced using FLASH (36). After truncating the Barcode and primer sequences, the splicing sequence obtained was raw tags data (Raw Tags). The Raw Tags obtained by splicing need to undergo a rigorous filtering process (37) to obtain high-quality tags data (Clean Tags). Furthermore, the final effective data (Effective Tags) are obtained by tags interception, tags length filtering, and removal of chimeric sequences. All Effective Tags of each sample were clustered by using the Uparse algorithm (Uparse v7.0.1001, http://www.drive5.com/uparse/) (38), and sequences that met the criteria of 97% consistency (Identity) and above were clustered into OTUs, and then the representative sequences of each OTU were filtered, and the representative sequences had the highest frequency of occurrence in their respective OTUs. The SSUrRNA database (39) using the Mothur method and SILVA138 (http://www.arb-silva.de/) (40) was used to annotate all representative sequences at each level row of related species (threshold set at 0.8–1) and then at each taxonomic unit level: kingdom, phylum, class, order, family, genus, and species to summarize the strain composition of each sample. Multiple sequences were then compared quickly by MUSCLE (41) (Version 3.8.31) software to obtain the relationships of the sequences represented by OTUs at each taxonomic level, and the OTUs information of each sample was homogenized by the minimum amount of data contained within each sample. Alpha diversity was calculated to determine the complexity of species diversity in each sample, and Observed-OTUs and Shannon index were calculated using QIIME (Version 1.7.0) software, and Venn diagrams, dilution curves, and cumulative box plots of species were plotted using R software (Version 2.15.3). Alpha diversity index analysis was used to obtain information on the differences between groups. *t* Test and non-parametric tests (Wilcoxon rank sum test) were used to compare bacterial abundance and diversity. Beta diversity calculations were analyzed using PCoA. Heat maps were constructed based on the non-parametric Wilcoxon test (*P* < 0.05 and *q* < 0.1) at the species and genus level. LEfSe analysis was performed by LEfSe software, and the default screening value of the LDA score was set to 3.0. Species with significant differences in composition between the two groups at each level were obtained by the *t* test and were also plotted and analyzed using R software. The function-based predictive analysis is performed by Tax4Fun based on the 16S rRNA OTU membership (42).

### Metabolomics

Hundred milligram of feces from each sample after liquid nitrogen grinding was added to 500 µL of 80% aqueous methanol in labeled tubes, shaken and mixed, then placed in an ice bath for 5 min, and centrifuged at 15,000 g for 10 min at 4°C. The supernatant was diluted to 53% methanol with mass spectrometry grade water and placed in a centrifuge tube at 15,000 g for 10 min at 4°C. UHPLC-MS/MS analysis was performed using a Vanquish UHPLC system in combination with a Q Exactive HF-X series mass spectrometer (Thermo Fisher). The sample was injected into a Hypesil Gold column at a flow rate of 0.2 mL/min. Mobile phase A (0.1% FA aqueous solution) and mobile phase B (methanol) are in positive polarity mode. Mobile phase A (5 mm ammonium acetate, pH 9.0) and mobile phase B (methanol) were in negative polarity mode. Solvent gradients were set as follows: 2% B, 1.5 min; 2%–100% B, 12.0 min; 100% B, 14.0 min; 2%–100% B, 14.1 min; 2% B, 17 min. The Q Exactive mass spectrometer was operated in both positive and negative polar modes. The spray voltage was set to 3.2 kV, and the capillary temperature was set to 320°C. The downstream data were imported into CD3.1 data processing software for data preprocessing, and the identification was made more accurate by the simple screening of parameters such as mass-to-charge ratio and retention time, followed by peak alignment for different samples based on retention time deviation of 0.2 min and mass deviation of 5 ppm. Peak extraction was performed according to the set information of mass deviation, signal intensity deviation, signal-to-noise ratio, minimum signal intensity, summed ions, etc. The same quantitative calculation of peak area was performed, and the peak area of each characteristic peak indicated the relative quantitative value of a metabolite. Next, the target ions were integrated, and then the molecular formula was predicted by molecular ion peaks and fragment ions and compared with mzCloud, mzVault, and Masslist databases. Background ions were removed using blank samples, and the quantitative results were normalized. Finally, we obtained the quantitative and identification results of metabolites.

### Based on LC-MS data analysis

The raw files (.raw) obtained by mass spectrometry were first imported into Compound Discoverer 3.1 software, and the quantitative and qualitative results of metabolites were obtained by spectrogram processing and database searching, followed by quality control to ensure the reliability and accuracy of data results. Annotation was performed using the KEGG database and the Human Metabolome Database. Metabolites were then subjected to PLS-DA multivariate statistical analysis, and statistical significance (*P*-value) was calculated using one-way analysis (*t* test). Metabolites with VIP > 1, *P*-value < 0.05, fold change ≥ 2, or FC ≤ 0.5 were considered to be different. Metabolite correlation analysis and hierarchical clustering were used to reveal the relationships between samples and between metabolites and metabolites. Ultimately, the biological significance of metabolite correlations was explained by functional analysis of metabolic pathways.

### Combined microbiome-metabolome analysis

Correlations between different flora and metabolites were calculated based on a one-to-one correspondence between samples. The correlation algorithm was performed using the Spearman correlation calculation method. *P* < 0.05 was considered statistically significant.

## Data Availability

The data sets provided in this study can be found in the online repository. The names and accession numbers of the repositories can be found at PRJNA960703.

## SUPPLEMENTAL MATERIAL

supplemental figures spectrum.04012-23-s0001.pdf Fig. S1 to S5.

Table S1 spectrum.04012-23-s0002.xlsx Basic clinical information of esophageal squamous cell carcinoma group and healthy control group.

Table S2 spectrum.04012-23-s0003.xlsx Quality control data of 16s rRNA gene sequencing.

Table S3 spectrum.04012-23-s0004.xlsx 48 discriminatory genera of bacteria.

Table S4 spectrum.04012-23-s0005.xlsx LC-MS-based differential metabolite abundance data for ESCC and HC group.

Table S5 spectrum.04012-23-s0006.xlsx Correlation analysis of differential metabolites.

Table S6 spectrum.04012-23-s0007.xlsx Results of KEGG enrichment analysis of differential metabolites between ESCC and HC groups.

Table S7 spectrum.04012-23-s0008.xlsx The Spearman relationship between 20 discriminative genera and metabolites in different classes.

ASM does not own the copyrights to Supplemental Material that may be linked to, or accessed through, an article. The authors have granted ASM a non-exclusive, world-wide license to publish the Supplemental Material files. Please contact the corresponding author directly for reuse.
