# Supplementary material for: Combining fecal microbiome and metabolomics reveals diagnostic biomarkers for esophageal squamous cell carcinoma
Source: Microbiol Spectr. 2024 Mar 18;12(5):e04012-23. doi: 10.1128/spectrum.04012-23 (PMC11064534; doi:10.1128/spectrum.04012-23)
Supplement: supplemental figures — Fig. S1 to S5. [file spectrum.04012-23-s0001.pdf]

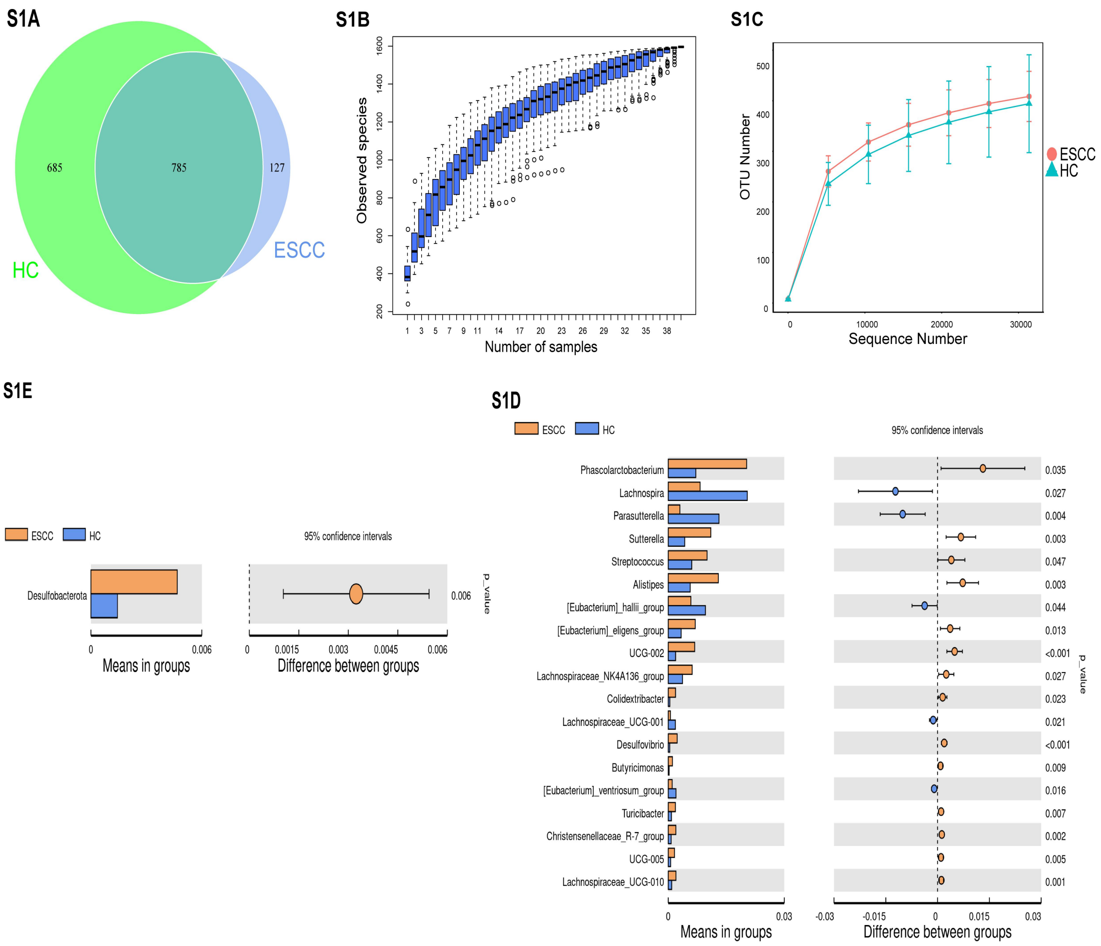

Fig.S1:Differential intestinal microorganisms between ESCC and HC groups. **(S1A):**912 OTUs and 1470 OTUs were identified in the ESCC and HC groups, respectively.**(S1B)**The species accumulation box plot can be used as a judgement of the adequacy of the sample size, the box plot tends to be flat, then the sampling is adequate.**(S1C)**The rarefaction curve directly reflects the reasonableness of the sequencing data volume and indirectly reflects the richness of species in the samples. In this study, the curve tends to be flat, indicating the reasonableness of the sequencing data volume.**(S1D， S1E)**The left panel shows the display of species abundance for intergroup differences at the phylum and genus levels. The right panel shows the between-group difference confidence display, and the leftmost point of each circle in the figure represents the lower limit of the 95% confidence interval for the difference in means, and the rightmost point of the circle represents the upper limit of the 95% confidence interval for the difference in means. The center of the circle represents the difference of means. The group represented by the color of the circle is the group with high mean. The rightmost end of the displayed results is the p-value of the between-group significance test corresponding to the difference species.

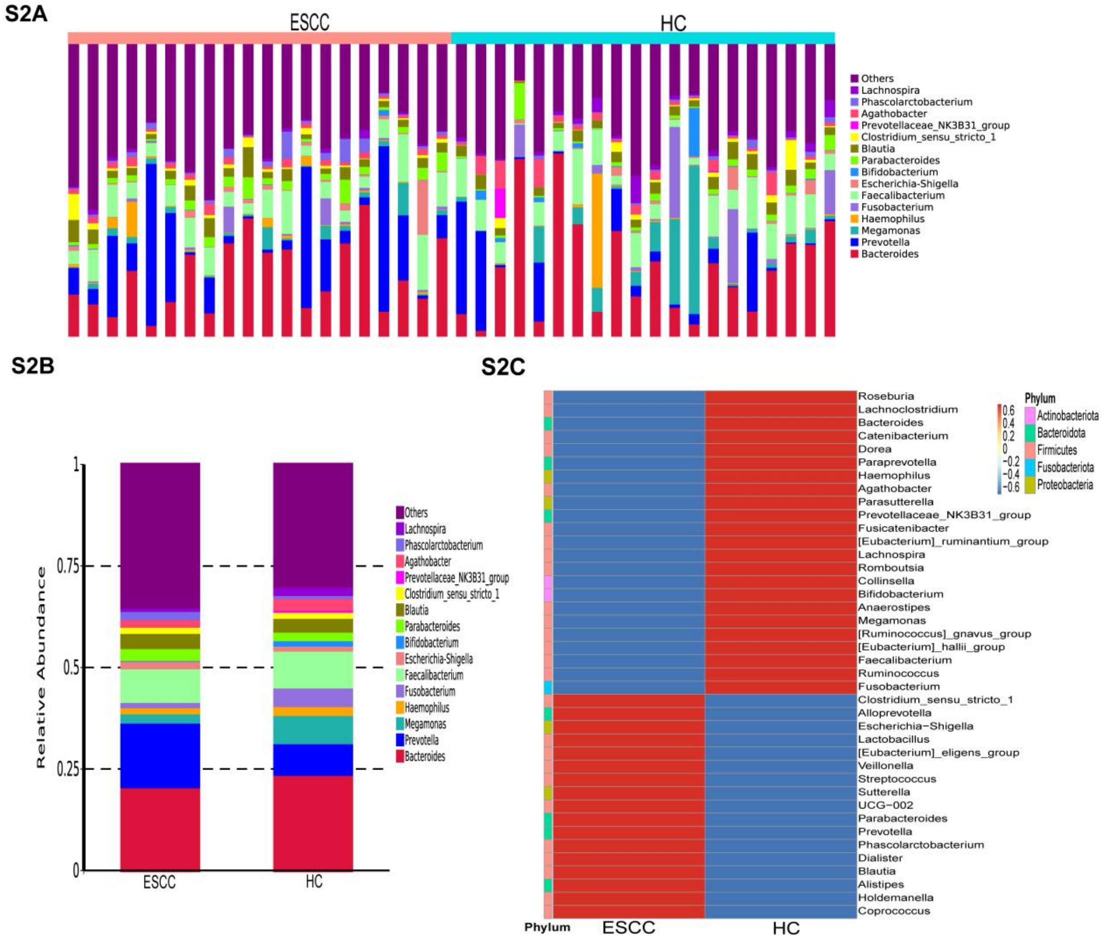

Fig.S2:Differential intestinal microorganisms at the genus level in the ESCC and HC groups.

(S2A,S2B):Proportions of bacterial genus levels in ESCC and HC groups. ESCC group:n=20,HC group:n=20. The vertical coordinate (Relative Abundance) indicates the relative abundance; Others indicates the sum of the relative abundance of all the other gates in the graph except these 10 gates.**(S2C):**The top 35 genera in terms of abundance were selected and clustered according to their abundance information in each sample, and plotted as a heat map.

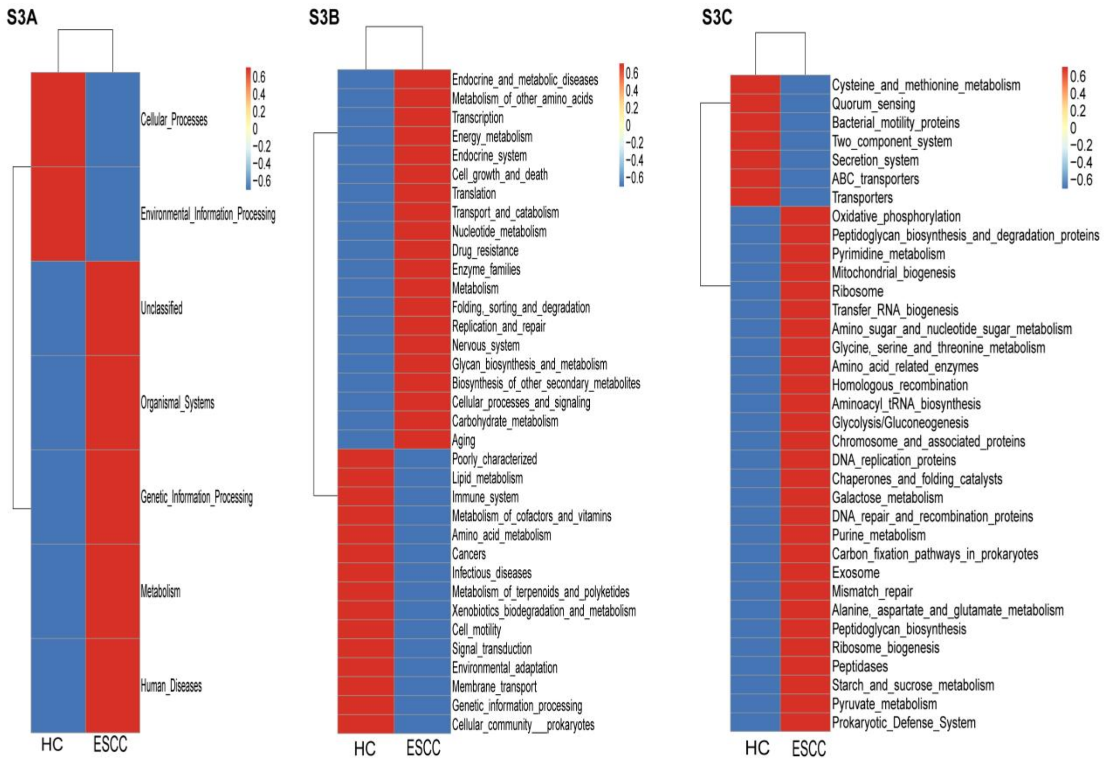

Fig.S3:Tax4Fun function predicts intestinal flora in ESCC and HC groups.

(S3A,S3B,S3C):Based on the functional annotations and abundance information of the two groups of samples in KEGG, the top 35 functions in terms of abundance and their abundance information in each sample were selected for heat map and clustered at the level of functional differences at levels 1, 2 and 3, respectively.

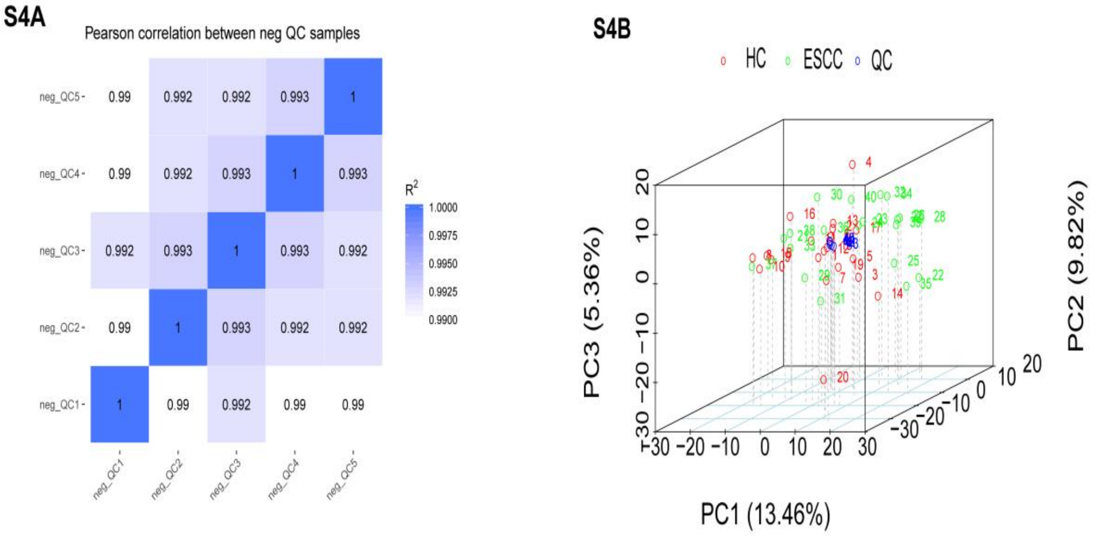

Fig.S4: Data quality control analysis of ESCC and HC group samples.

(S4A): Quality control QC (Quality Control) is to ensure the reliability of data quality and exclude the interference of some external factors. The relative quantitative values of metabolites are used to calculate the Pearson correlation coefficient between QC samples. The closer the R2 is to 1, the higher the correlation is, the more stable the whole assay process is, and the more accurate and reliable the data results are. **(S4B)** PCA, as a non-parametric method, can reduce the dimensional representation of the provided raw data to obtain new feature variables.The horizontal coordinate PC1 in the figure indicates the score of the first ranked principal component, and the vertical coordinate PC2 indicates the score of the second ranked principal component, respectively, and the color of the dots is used to distinguish different groupings.

S5

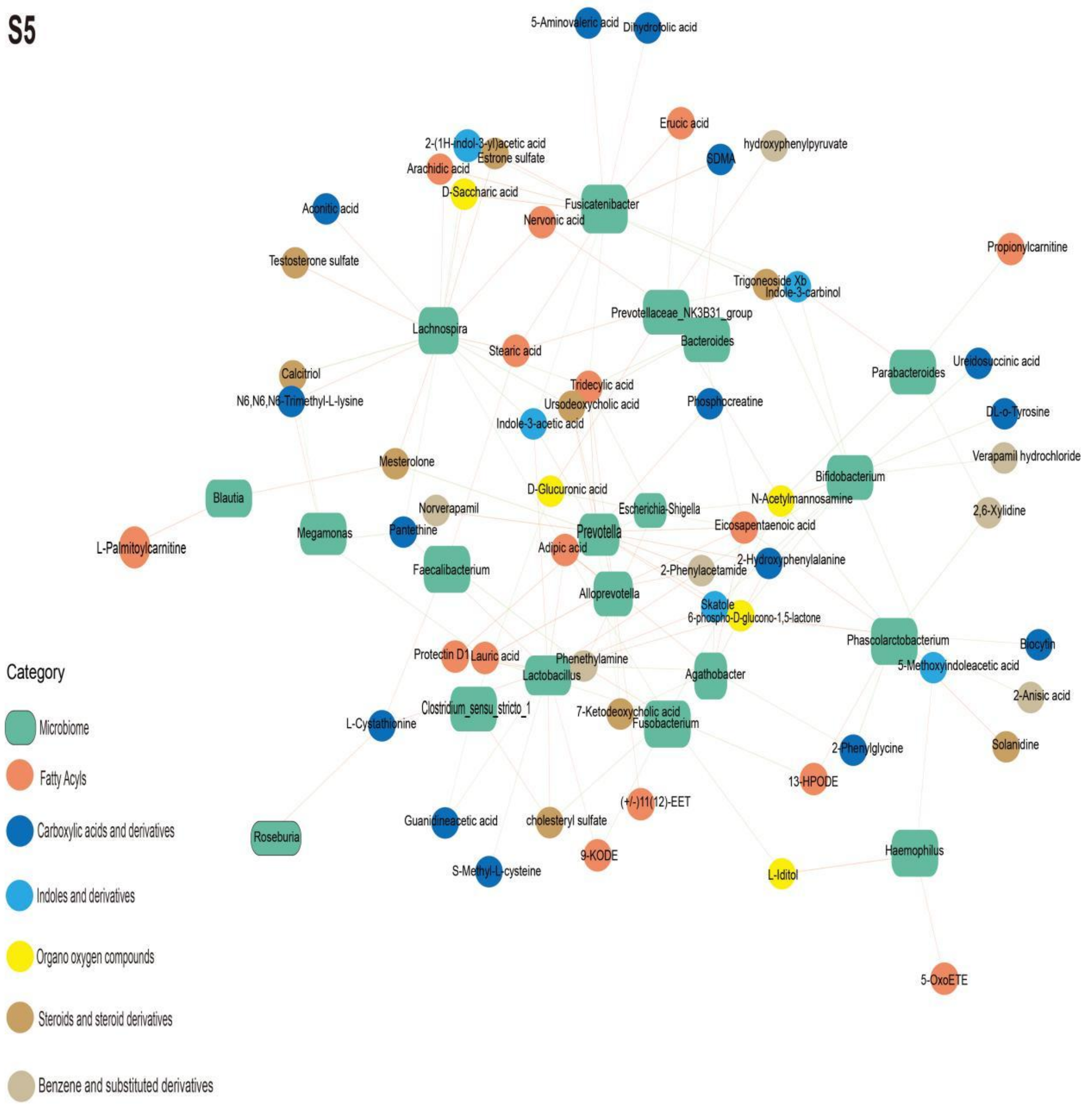

Fig.S5: Network diagram based on spearman correlation analysis to calculate the correlation between species and metabolite data, selected pairs of relationships with pvalue <= 0.05 were plotted. The red line represents positive correlation and the green line represents negative correlation. The thickness of the line represents the correlation coefficient
